# The level of cognitive functioning in school-aged children is predicted by resting EEG Directed Phase Lag Index

**DOI:** 10.1038/s41598-025-85635-6

**Published:** 2025-01-09

**Authors:** Audrey-Rose Charlebois-Poirier, Saeideh Davoudi, Ève Lalancette, Inga Sophia Knoth, Sarah Lippé

**Affiliations:** 1 Research Center of the Centre Hospitalier Universitaire Sainte-Justine, Montreal, QC Canada; 2 Department of Psychology, University of Montréal, Montreal, QC Canada; 3 Department of Neuroscience, University of Montréal, Montreal, QC Canada

**Keywords:** Cognitive functioning, Electroencephalography, Multiscale entropy, Directed Phase Lag Index, Brain development, Cognitive neuroscience, Neural circuits, Neuroscience

## Abstract

Quantifying cognitive potential relies on psychometric measures that do not directly reflect cortical activity. While the relationship between cognitive ability and resting state EEG signal dynamics has been extensively studied in children with below-average cognitive performances, there remains a paucity of research focusing on individuals with normal to above-average cognitive functioning. This study aimed to elucidate the resting EEG dynamics in children aged four to 12 years across normal to above-average cognitive potential. Our findings indicate that signal complexity, as measured by Multiscale Entropy (MSE), was not significantly predictive of the level of cognitive functioning. However, utilizing Directed Phase Lag Index (DPLI) as an effective connectivity measure, we observed consistent patterns of information flow between anterior and posterior regions. Fronto-parietal as well as local connectivity patterns were seen across most of the cognitive functions. Moreover, specific connectivity patterns were obtained for each intellectual quotient index (namely verbal comprehension, visuospatial, fluid reasoning, and processing speed indexes as well as full-scale intellectual quotient). These results underscore the presence of long-range connections and support fronto-parietal theories of cognitive abilities within the resting state brain dynamics of children.

## Introduction

The brain’s functional organization, efficiency, and capacity for information processing, are critical components of intelligence^1–3^. In recent years, several EEG metrics have been shown to reflect the brain’s functional organization, including when the brain is at rest^4,5^. Resting-state electroencephalography (RS-EEG) allows us to study the brain’s intrinsic activity so that we record the temporal dynamics while sensory information from the environment gets to freely interact with brain systems^6–8^. Studies have shown that RS-EEG metrics are related to various cognitive functions^9^. Indeed, resting state networks are activated when performing tasks and subserve functions like learning and adaptation to environmental uncertainty^10^. This suggests that resting state activity metrics could be related to the level of cognitive ability as measured by psychometric tests.

The interest in studying resting state cortical dynamics as measured by EEG in above-average intelligence children comes from the different theoretical models explaining the potential sources of their cognitive talents. Firstly, Richard Haier proposed his Neural Efficiency Theory, by which individuals with higher-than-average cognitive performances showed an efficient use of their cortical energy. He showed that these individuals used less blood glucose than their peers with cognitive abilities within the average of their age group^11–13^. In link with this theory, the Parieto-Frontal Integration theory came up with a potential explanation for this reduced glucose use. Indeed, the authors proposed that individuals with higher cognitive ability had a more targeted and rapid use of brain regions necessary to complete a task. This rapid targeting of important brain regions could be linked with the perfect excitation/inhibition balance that is found in the resting brains of children with high cognitive ability. This excitation/inhibition (E/I) balance reflects the maturation of GABAergic inhibitory circuitry which facilitates the experience-dependent pruning of excitatory neurons^14^. Therefore, an important feature of cognitive development is a reduction in the E/I ratio with age^15^. A recent fMRI study showed that among children with the same chronological age, a lower E/I ratio was linked to better cognitive performance^16^. Further, modulations in E/I ratios have been associated with changes in resting state cortical dynamics. Indeed, shifts towards inhibition were linked with vigilance impairment versus increased excitation lead to network hyperexcitability^17^. Therefore, studying the relationship between cortical dynamics at rest and intellectual potential in children is based on both theoretical and biophysiological concepts.

Over the last decades, RS-EEG signal complexity was investigated as a mechanism underlying a system’s ability to be dynamic, and adaptive to its environment^5,8,18^. Multi-scale entropy (MSE) is a measure of signal complexity that shows the amount of similarity across a time series (e.g., EEG signal). MSE reflects the degree to which a signal is predictable over progressively longer time segments, where smaller values reflect repetitive fluctuations^10,19^. In most systems, higher complexity values are associated with efficient functioning^20^. Complexity values increase with age and correlate with the system’s level of maturation. It is believed to increase with age because brain regions become more specialized and differentiated, allowing for a wider range of neural dynamics^21,22^. The relationship between brain complexity and age follows an inverted U-curve, where increased complexity (i.e., higher MSE) is observed from childhood to late adolescence, and a decrease is seen in old age due to normal aging^5,10^. MSE values are also impacted in many neuropsychiatric conditions and genetic syndromes, where deficits in cognitive functioning are observed^5,23–26^. Similarly, MSE studies performed on adults with intellectual disability demonstrated a positive relationship between reaction times on a simple visual task and signal complexity (i.e. slower reaction times were related to less complexity)^27^. These studies therefore show that EEG signal complexity is related to brain development and is impacted in the lower-than-average range of cognitive ability. While holding for the population with a lower than average and average intellectual abilities, it is not clear the rational holds true for children with above average levels of cognitive ability.

While complexity allows us to understand the richness of the neural networks^18^, connectivity measures reflect the coordination of brain regions underlying the generation of representations^28^. With development, the brain shows increased front-parietal connections, which are crucial for information processing efficiency within the brain^29^. Additionally, using the measure of phase slope index, Thatcher et al.^30^ found that individuals with higher cognitive performances on intelligence tests (i.e. FSIQ) showed more efficient information processing as defined by greater connectivity between frontal and parietal regions and reduced demand on long-distance connections (i.e. fronto-occipital connections).

Increasing evidence suggests that resting cortical activity follows a directional pattern between brain regions^31^. In contrast to functional connectivity, which only gives information about the temporal correlations^32^, directed phase-lag index (dPLI)^33^ is a measure of effective connectivity that defines the magnitude and direction of information flow between two or more connected brain regions^30^. More precisely, dPLI values convey the extent to which one cortical region is leading or lagging in phase compared to other regions of the brain^31^. Recent magnetoencephalography studies reported that cortical information flow during rest follows a posterior to anterior pattern for high-frequency bands (i.e. alpha, beta, and gamma) which reverses for low-frequency bands (delta and theta) and that this pattern was associated with better cognitive flexibility^34^. No study to our knowledge has studied dPLI measures in relation to general cognitive ability using EEG, and in school-aged children.

Furthermore, children with above-average intelligence have been overlooked in scientific studies investigating directed connectivity. The direction of the connections between complex neuronal networks can inform how the brain is organized to support information integration and segregation^35^. In the context of above-average intelligence, changes in dPLI can reflect the progression of information flow with respect to general cognitive ability. These patterns of brain dynamics can also help identify directions of information flow that lead to efficient processing of the environment at rest.

Considering that cognitive ability is defined as an individual’s ability to adapt and constructively respond to their environment’s demands^36–40^, and that RS brain dynamics reflect the brains integration of its surrounding environment, this then raises the question if RS EEG metrics can predict above average cognitive ability in school-aged children. Although other neuroimaging techniques (e.g. fMRI) have approached the question, as demonstrated by Vieira et al.^41^’s view^41^, to our knowledge, our study is the first to explore the link between above-average cognitive ability and RS EEG complexity in children. Furthermore, the direction of connectivity patterns during rest has never been explored in the context of cognitive ability in children. Therefore, this research is the first to use the innovative measure of dPLI to investigate the link between RS-EEG dynamics and the level of cognitive functioning in school-aged children.

In this study, we investigated the complexity and effective connectivity of the resting brain’s signal in children aged from four to 12 years. We used MSE as a measure of complexity and dPLI to quantify effective connectivity. We expected to observe a different organization of the resting brain with higher levels of cognitive ability, as measured by psychometric intelligence tests. Firstly, based on the findings reported above, we expected to see increasing RS-EEG complexity in all time scales with increasing levels of cognitive functioning. Secondly, we hypothesized that more fronto-parietal and fronto-occipital connections would be observed with increasing levels of cognitive functioning. This investigation would help further our understanding of the relationship between above-average cognitive ability and resting state dynamics.

## Methods

### Participants

**Fig. 1:**
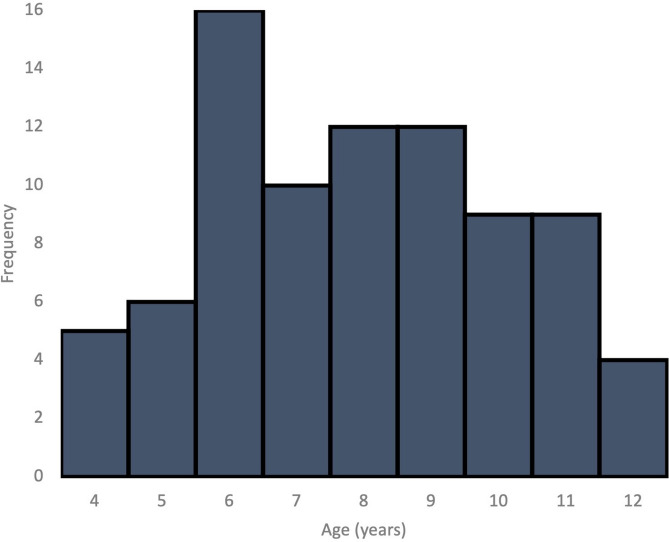
Age distribution of the 83 participants included in the analysis.

Resting state EEG and neuropsychological testing were performed for ninety children. Seven children had to be excluded from the EEG analyses due to movement artifacts or neuropsychological results within clinical thresholds (see the following section for further details on neuropsychological testing). The remaining 83 participants (50 males) ranged in age between four and 12 years ($\overline{X }$=7.94 years, SD = 2.254 years; age distribution is shown in Fig. 1). Children were recruited using ads, posts on social media, and emails to parents in specialized elementary schools within the Centre des Services Scolaires de Montréal. Exclusion criteria included a history of brain injury as well as psychiatric, neurological, and/or neurodevelopmental disorders. Participants of all ethnic and socioeconomic backgrounds were encouraged to participate. The study protocol was reviewed and approved by the ethics, administrative, and scientific committee of the CHU Ste-Justine Hospital Research Center and follows the declaration of Helsinki principles. Procedures undertaken were explained to participants and parents, and written informed consent was obtained from both the parents and children (when possible).

### Cognitive ability evaluation

Cognitive ability evaluations were performed using the Weschler Preschool and Primary Scale of Intelligence fourth edition (WPPSI-IV) or the Weschler Intelligence Scale for Children fifth edition [WISC-V^42^] depending on the participant’s age. All mandatory subtests to obtain the FSIQ and primary index scores (i.e. verbal comprehension (VCI), visual-spatial (VSI), fluid reasoning (FRI), processing speed (PSI), and working memory (WMI) indexes) were administered. The Code Transmission subtest from the Test of Everyday Attention for Children [TEA-Ch^43^] was administered to identify subthreshold performances in sustained attention and to help identify potential attention disorders. The California Verbal Learning Test for Children [CVLT-C^44^] was administered to evaluate verbal learning and memory capacities, to identify children who may have learning disorders. Scores to these neuropsychological tests (i.e. code transmission and CVLT-C) were transformed into percentiles using norms for age and biological sex. Aberrant results were defined as those under the clinical threshold of percentile two and lower. One participant was removed after this step. Table 1 shows the results obtained for the 83 participants for all neuropsychological tests considered for this cohort.

**Table 1:** Description of results on neuropsychological measure.

Neuropsychological measure	Min–max	Mean (± std. dev)
FSIQ	85–139	114 ± 12
VCI	83–146	114 ± 14
VSI	63–144	111 ± 17
FRI	69–140	112 ± 12
WMI	76–138	107 ± 15
PSI	72–144	101 ± 14
TEA-Ch code transmission	1–18	10.17 ± 3.814
CVLT-C recognition hits	− 2.5 to 11	0.548 ± 1.525

Average scores for all IQ indexes and neuropsychological tests for all included participants. IQ subscale units (FSIQ, VCI, VSI, FRI, WMI, and PSI) are reported as age-standardized composite scores while neuropsychological test (TEA-Ch and CVLT-C) scores are reported as age-corrected Standard scores and Z-scores respectively.

To further ensure the absence of neurodevelopmental comorbidities in the participant cohort, parents were asked to fill out questionnaires regarding their child’s development and abilities. As attentional deficit disorders can often be observed in children with high IQs (i.e. twice-exceptionality, (Minahim & Rohde, 2015)), these comorbidities were evaluated using the Conners-3^45^. Scores below the second percentile inclusively, indicate the presence of ADHD symptoms. Potential behavioral and emotional problems were identified using the Child-Behavior-Checklist [CBCL^46^]. Scores above the 98th percentile indicate that the child may have attentional deficits. Participants demonstrating results that exceeded the clinical threshold mentioned above were removed from the analysis as we might suspect undiagnosed neurodevelopmental conditions. One participant was removed from the analysis for obtaining clinical thresholds for inattentiveness and hyperactivity on the CBCL and the Conners. Table 2 describes the scores for each attention deficit symptom scale.

**Table 2:** Description of results on questionnaires.

Questionnaire scale	Min–max (t-score)	Mean (± std. dev)
Conners 3—DSM-IV ADHD inattentive scale	40–84	52.41 ± 9.573
Conners 3—DSM-IV ADHD hyperactive/impulsive scale	40–90	52.22 ± 11.37
CBCL—attention problems scale	50–70	63.86 ± 5.00

The span of scores for attention problem scales of the CBCL and ADHD scales of the Conners 3 questionnaires for the 83 children included in this study.

### Data acquisition and preprocessing

EEG was recorded in an electrically shielded and dark soundproof experimental chamber. A research assistant and/or parent (depending on the participant’s needs) was present in the room as the recording took place. A high-density EEG system containing 128 electrodes was used for continuous recording (Electrical Geodesics System Inc., Eugene, OR, USA) of a 3-min eyes open and a 2-min eyes closed resting period. To limit movement artifacts, children were told to look at a screen showing a muted movie without subtitles during the eyes open resting recording, as previously done in many studies with children^23,47,48^. Signals were acquired using an EGI Net Amp 300 amplifier and saved on a G4 Macintosh computer using NetStation EEG Software (Version 4.5.4). Data were acquired at a 1000 Hz sampling rate and an analog bandpass filter of 0.1–500 Hz was applied. The vertex was used as the reference electrode during recording and impedances were maintained below 40 kΩ^49^. Offline pre-processing was carried out with MATLAB (version R2023a) and EEGLAB toolbox (v.14.1.1). Data were digitally filtered with a lower bound of 0.5 Hz and a higher bound of 150 Hz and a 60 Hz notch filter. The same twenty-eight electrodes containing muscular artifacts, around the neck and face were removed for all participants (see Fig. 3). Electrodes with a total standard deviation lower than 2 μV and higher than 200 μV were removed automatically. Channels with sporadic behavior were then manually removed during subsequent visual inspection. As a result, an average of 1.45 electrodes (± 2.351 electrodes) was removed for each participant. Data was then re-referenced to an average reference. Eye movement and cardiac artifacts were corrected using the semi-automatic independent component analysis tool implemented in EEGLAB. Continuous data was segmented into four-second intervals and epochs with spontaneous voltage amplitudes of ± 200 μV were automatically marked and removed. Finally, all trials were visually inspected and those contaminated with artifacts were manually removed. The average percentage of epochs removed per participant was 22.09%.

**Fig. 2:**
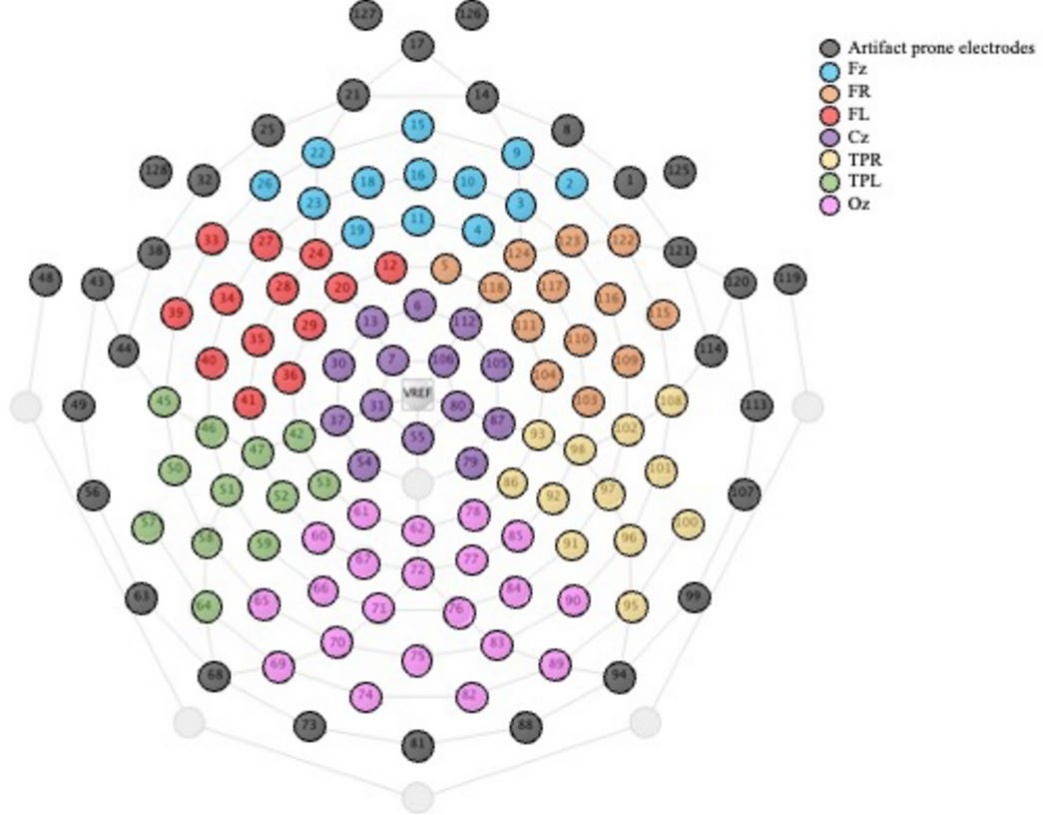
Electrode map of the regions of interest. The electrodes were divided for each region of interest.

To understand the role of each brain region, the electrode net was divided into seven regions of interest (ROIs) based on anatomical brain regions: Frontal (Fz), Frontal right (FR), Frontal left (FL), Central (Cz), Temporoparietal right (TPR), Temporoparietal left (TPL) and Occipital (Oz). Each region was made up of clusters of electrodes on the 128-electrode net as illustrated in Fig. 2.

### Complexity as measured by multi-scale entropy

To determine the regularity of the resting EEG signal (i.e. the number of repetitions of patterns in the system’s trajectory), multi-scale entropy (MSE) calculations were applied to the preprocessed data^5,8^. MSE calculations were based on the methods used in Proteau-Lemieux et al.^23^ and Davoudi et al.^8^, which followed the algorithm presented by Costa et al.^50^. This method calculates sample entropy at multiple time scales of the EEG signal using a coarse-graining procedure. The procedure involves the down-sampling of the original time series, where for a time scale τ, consecutive points are averaged across nonoverlapping windows of length τ. Therefore, the time series shorten as the window length increases. Sample entropy (SE) was then obtained for each coarse-grained time series. In this paper, MSE was estimated in 40 temporal scales in each electrode as well as epoch and then averaged across epochs for each participant^8,23^.

Given the original time series with *N* samples [*x*(1), *x*(2), *x*(3), …, *x*(*N*)], averaging neighboring points formed the coarse-grained time series for scale τ:

1 $${y}_{j}^{(\uptau )}=\frac{1}{\uptau }{\sum }_{i=\left(j-1\right)\uptau +1}^{j\uptau }x(i), 1\le j\le \frac{N}{\uptau }$$

SE for a given embedding pattern length *m*, tolerance *r*, and number of data points *N* was calculated for each given scale factor using the following:

2 $${S}_{E}\left(m, r, N\right)= -\text{ln}\left(\frac{{C}_{m+1}(r)}{{C}_{m}(r)}\right)$$

where, ${C}_{m}$ is the probability that points *m* distance apart would be within the distance *r. m* was set at two (meaning that the algorithm counts the number of matching sequences for two consecutive points in the signal) and *r* was set at 0.5 (indicating that amplitude points falling ≤ 50% of the time-series’ standard deviation were considered by the algorithm) based on Kosciessa et al.^19^.

MSE values for all epochs were averaged for each participant and region of interest to obtain a final MSE score for every time scale from 1 to 40. Following the MSE calculation as well as the plotting of the SE for each scale against the temporal scales, the area under the curve is calculated. This value is then considered to be the Complexity index (CI):

3 $$Complexity Index \left(CI\right)={\sum }_{i=1}^{T}{S}_{E}\left(i\right)$$

where *T* is the total number of scales.

Since low and high scales are believed to be associated with different EEG oscillatory patterns^51^, and for data reduction purposes, the CI for each scale within one to 20, 21 to 40, and 1 to 40 were averaged together. Therefore, each participant had three CI values per region of interest.

### Directed Phase-Lag Index to measure connectivity

Directed connectivity was measured using Directed Phase Lag Index (dPLI), which is defined as the probability that the instantaneous phase of X is smaller than the phase of Y (modulo π)^33^. Unlike PLI which only measures the non-zero phase synchronization between two signals regardless of the direction of information, dPLI is a modified PLI indicating which of two signals is lagging and which is leading in phase. Therefore, unlike PLI which only measures the strength of the relationship between two brain signals, DPLI specifies the strength of the direction of the information. DPLI values are bounded by 0 to 1; whereas time series $x$ is said to phase-lead time series $y$ if $0.5<{dPLI}_{xy}\le 1$ and $x$ is phase lagging compared to $y$, when $0.5\le {dPLI}_{xy}<0.5$. If neither $x$ nor $y$ is leading or lagging on average, then ${dPLI}_{xy}=0.5$. If we assume ${\phi }_{t}={{\phi }_{x}-\phi }_{y} (t=1$… *N)*, in which ${\phi }_{x}$ and ${\phi }_{y}$ show the instantaneous phase of time series x and time series y, respectively, the dPLI of time series x with respect to time series y can be defined as the probability $\phi \left(t\right)>0.$

4 $${dPLI}_{xy}=\frac{1}{N} {\sum }_{t=1}^{N}H\left({\phi }_{t}\right)$$

where *H* is the Heaviside step function.

DPLI is robust for volume conduction and reference montages. In this paper, the dPLI values were calculated for each 4-s epoch, across all pairs of electrodes, within each frequency band and for each participant, The frequency bands were: delta (1–3.5 Hz), theta (4–7.5 Hz), alpha (8–12 Hz), beta (13–29 Hz), low-gamma (30–49 Hz), high-gamma (50–70 Hz) and broadband (1-70 Hz). These dPLI values were then averaged across all epochs, resulting in a matrix with dimensions corresponding to electrode x electrode for each frequency band and for each participant DPLI was calculated for each connection between ROI, resulting in 21 possible connections. The dPLI values were statistically corrected with surrogate data to avoid spurious connections. Using a surrogate distribution is a robust statistical approach that enhances the reliability and validity of our results by addressing potential confounding factors inherent in EEG data. Such factors, which can include noise, volume conduction and field spread effects, can lead to spurious correlations between electrodes that do not represent genuine neural interactions. To mitigate this issue, we generated surrogate data by systematically shuffling the original EEG data—while preserving certain statistical properties, such as amplitude—and randomizing the phase. This process disrupts any false connectivity, providing a baseline measure of connectivity. By comparing the observed connectivity values with the surrogate distribution, we can distinguish genuine neural connections from those that might arise due to noise, volume conduction, or chance. 500 surrogate distributions were generated by arbitrary time lags between ${\phi }_{x}(t)$ and ${\phi }_{y}(t),$ and the significant dPLI was chosen with a p-value of 0.05 according to the random surrogate distributions. This computation was performed using the built-in Matlab tollbox *NeuroAlgo*.

### Feature ranking and regression models

**Fig. 3:**
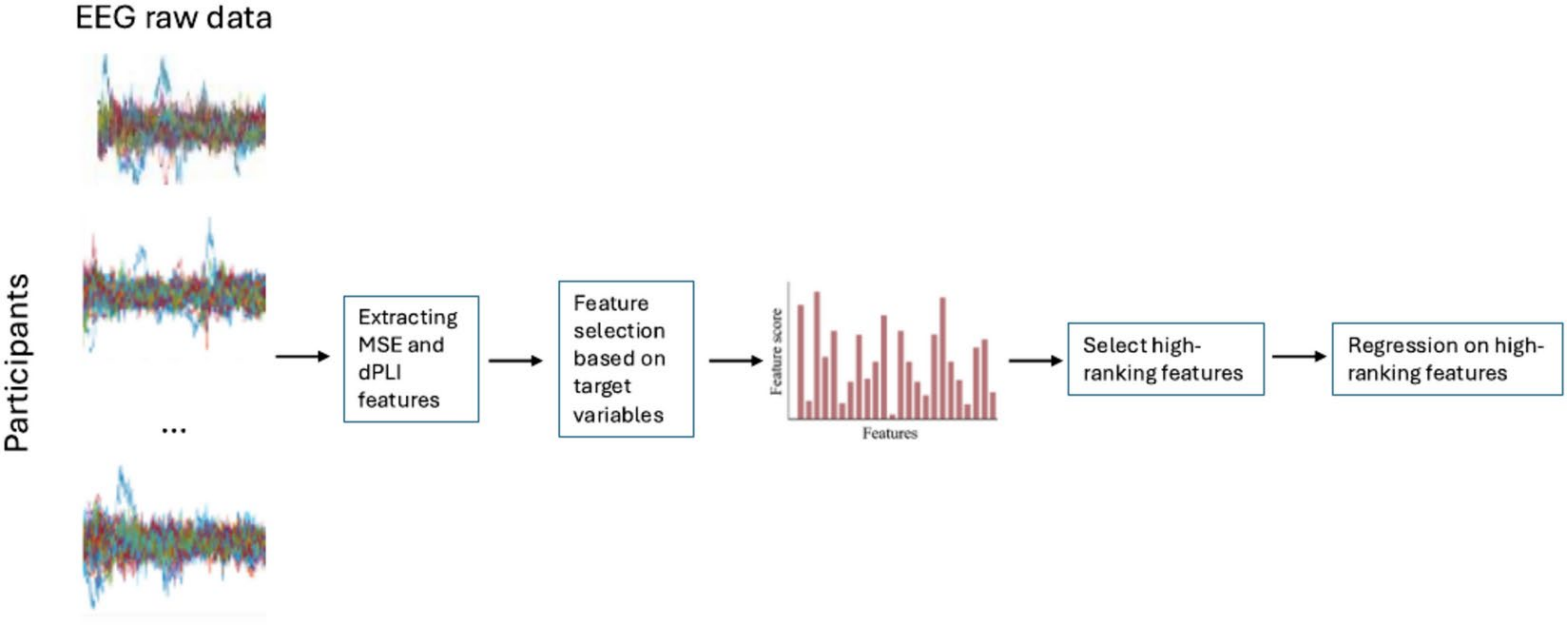
Schematic representation of Feature Ranking and Regression models method. The steps for Feature Ranking and Regression models method.

Univariate feature ranking for regressions based on F-tests was used to discern the individual relevance of each EEG feature and to determine its predictive value for each IQ index. When applying this method, each feature is assigned a rank, based on the F-statistic and associated p-value which reflect its contribution to the regression model. Indeed, for each feature, the F-test assessed how much the feature contributed to predicting the target variable, relative to a model without predictors. This method differs from an ANOVA based method, which is typically used for feature selection when the target variable is categorical. With the ranked list of features, we proceeded to implement a progressive feature selection strategy. Through a loop, the features were gradually added into the regression model, starting with the highest-ranked feature, and progressively incorporating additional features in the subsequent iterations of the loop. For each iteration, the impact of a newly added feature on the predictive performance of the model was monitored to observe how this performance evolved as more features were integrated. Figure 3 offers a schematic representation of the steps involved in the statistical method.

Twelve feature ranking analyses were run separately, one per EEG metric (MSE or dPLI), one per resting condition (eyes open or eyes closed), and per IQ index (VCI, VSI, FRI, WMI, PSI, and FSIQ). For the MSE models, a feature is a combination of one of the three scale averages (1–20, 21–40, or 1–40) with one of the seven regions of interest, totaling a maximum of 21 features that could be added to a model. Similarly, for the dPLI models, a maximum of 147 features could be added to the models (21 connections x seven frequency bands). Age and biological sex were added as control variables in this study.

## Results

Feature ranking was used to reduce the dimensionality of the multivariate regressions by adding the most predictive features in the models based on rank. This allowed us to optimize the models’ predictive abilities while targeting the most important EEG features for the IQ indexes. All models were controlled for the effects of age and biological sexes.

### Multiscale entropy

Of all the MSE models generated, the only significant models were related to VCI. The maximal performance model was obtained by using the 10 highest-ranking features for the eyes-closed condition and the nine highest-ranking features for the eyes-open condition. Within the model, the only significantly predictive feature was age, for both conditions (see Table 3 for statistical results). No significant model was found for any other IQ index.

**Table 3:** Significant statistical model for MSE.

	Eyes open				Eyes closed			
Model R^2^	0.183				0.199			
Model F	2.07				2.02			
Model *p*	0.0492				0.0489			
	**Sig. predictor**	**Estimate**	**SE**	***p***	**Sig. predictor**	**Estimate**	**SE**	***p***
	Age	2.304	0.770	0.00377	Age	2.13	0.815	0.0109

whole model R^2^ and p-values for the predictive value to VCI for each resting condition. Parameters for the significant predictive features are also presented per resting condition.

### Directed Phase Lag Index

**Fig. 4:**
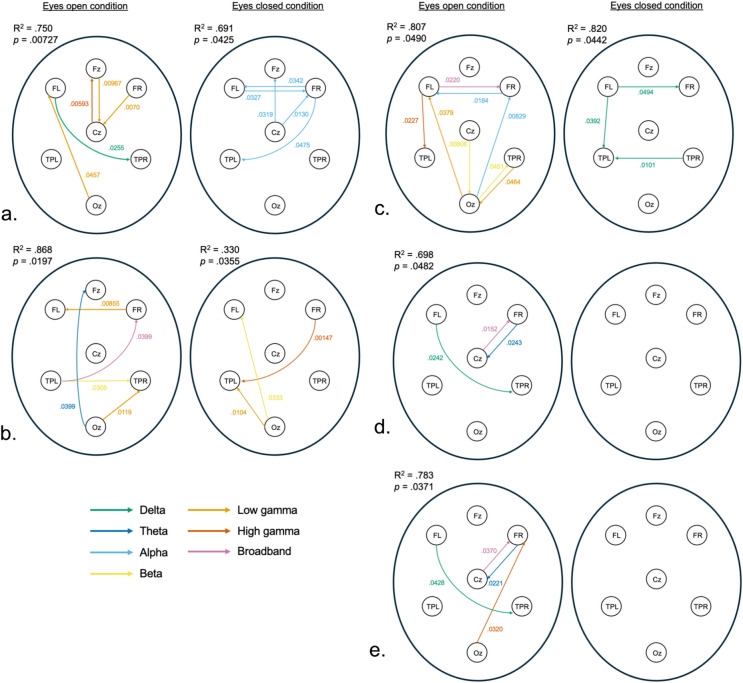
Schematic representation of significant dPLI results. Each panel represents the significantly predictive dPLI EEG features per IQ measure. Each frequency band is represented by a colour as explained by the legend. The whole model R^2^ and p-value are reported in the upper left portion of each map. Panel a.: VCI; b.: VSI; c. PSI; d. FRI; and e. FSIQ. Significant p-value per dPLI is reported in the corresponding frequency band colour.

The following sections summarize the significant predictors for each IQ measure. A visual representation of the significant predictive connections can be found in Fig. 4.

### Verbal Comprehension Index (VCI)

For VCI, significant models were obtained for both eyes open and eyes closed conditions. In both conditions, age was a significant control variable. For the eyes open condition, the selected model with the highest R^2^ contained 47 EEG features, while only five reached significance for the prediction of VCI. Similarly, the eyes closed condition final model had 46 EEG features, with only five reaching significance as predictors for VCI. See Table 4 for statistical results.

**Table 4:** Significant statistical model for VCI.

	Eyes open				Eyes closed			
Model R^2^	0.750				0.691			
Model F	2.24				1.75			
Model *p*	0.00727				0.0425			
	**Sig. predictor**	**Estimate**	**SE**	***p***	**Sig. predictor**	**Estimate**	**SE**	***p***
	Delta FL-TPR	4.279	1.834	0.0255	Alpha Cz-Fz	13.136	5.88	0.0319
	Low gamma Oz-FL	7.490	3.614	0.0457	Alpha FR-Fz	10.419	4.69	0.0327
	Low Gamma FR-Cz	9.145	3.192	0.0070	Alpha FL-FR	17.104	7.77	0.0342
	Low gamma Fz-Cz	11.963	4.370	0.00967	Alpha FR-TPL	10.956	5.34	0.0475
	High Gamma Cz-Fz	12.736	4.347	0.00593	Alpha Cz-FR	16.532	6.32	0.0130
	Age	1.834	0.744	0.0188	Age	2.537	0.938	0.0104

Whole model R^2^ and p-values for the predictive value to VCI for each resting condition. Parameters for the significant predictive features are also presented per resting condition.

### Visuospatial Index (VSI)

Significant VSI final models were also obtained for both resting conditions. The eyes open condition significant model had 61 EEG features with five being significant predictors for VSI, and the eyes closed had 17 predictors, four of which reached significance as predictors for VSI. See Table 5 for statistical results.

**Table 5:** Significant statistical model for VSI.

	Eyes open				Eyes closed			
Model R^2^	0.868				0.330			
Model F	2.27				1.89			
Model *p*	0.0197				0.0355			
	**Sig. predictor**	**Estimate**	**SE**	***p***	**Sig. predictor**	**Estimate**	**SE**	***p***
	Broadband TPL-FR	9.300	4.24	0.0399	Beta Oz-FL	5.805	2.67	0.0333
	Theta Oz-Fz	11.994	5.106	0.0287	Low gamma Oz-TPL	6.91	2.62	0.0104
	Beta TPL-TPR	12.192	5.256	0.0305	High gamma FR-TPL	11.004	3.31	0.00147
	Low gamma FR-Fz	14.373	4.955	0.00855				
	Low gamma Oz-TPR	13.152	4.777	0.0119				

Whole model R^2^ and p-values for the predictive value to VSI for each resting condition. Parameters for the significant predictive features are also presented per resting condition.

### Fluid Reasoning Index (FRI)

FRI models only reached significance for the eyes open resting condition. This final model contained 47 EEG features, three of which were significant predictors for FRI. No resting eyes closed EEG feature was a significant predictor for FRI. See Table 6 for statistical results.

**Table 6:** Significant statistical model for FRI.

	Eyes open			
Model R^2^	0.698			
Model F	1.72			
Model *p*	0.0482			
	**Sig. predictor**	**Estimate**	**SE**	***p***
	Broadband Cz-FR	7.969	3.120	0.0152
	Theta FR-Cz	6.437	2.735	0.0243
	Delta FL-TPR	6.223	2.642	0.0242

Whole model R^2^ and p-values for the predictive value to FRI for the eyes open resting condition. Parameters for the significant predictive features are also presented.

### Working Memory Index (WMI)

WMI models did not reach significance for either resting condition. No EEG feature had predictive value for WMI.

### Processing Speed Index (PSI)

For the PSI, significant models were obtained for both resting conditions. The eyes open condition model had a total of 57 EEG features, with eight being significant predictors for PSI. The eyes closed condition model contained 58 EEG features with only three reaching significance as predictors for PSI. See Table 7 for statistical results.

**Table 7:** Significant statistical model for PSI.

	Eyes open				Eyes closed			
Model R^2^	0.807				0.820			
Model F	1.83				1.89			
Model *p*	0.049				0.0442			
	**Sig. predictor**	**Estimate**	**SE**	***p***	**Sig. predictor**	**Estimate**	**SE**	***p***
	Broadband FL-FR	18.289	7.49	0.0220	Delta TPR-TPL	13.623	4.88	0.0101
	Alpha Oz-FR	23.037	8.036	0.00829	Delta FL-TPL	9.336	4.281	0.0392
	Alpha FR-FL	17.39	6.89	0.0184	Delta FL-FR	9.604	4.640	0.0494
	Beta Cz-Oz	11.775	4.09	0.00806				
	Beta Oz-TPR	9.189	4.357	0.0451				
	Low gamma Oz-FL	23.915	10.85	0.0370				
	Low gamma TPR-Oz	14.626	6.979	0.0464				
	High gamma FL-TPL	12.524	5.15	0.0227				

Whole model R^2^ and p-values for the predictive value to PSI for each resting condition. Parameters for the significant predictive features are also presented per resting condition.

### Full-Scale Intellectual Quotient (FSIQ)

Finally, the FSIQ models were only significant in the eyes open resting condition. This model included 54 EEG features, four of which were significant predictors for FSIQ. See Table 8 for statistical results.

**Table 8:** Significant statistical model for FSIQ.

	Eyes open			
Model R^2^	0.783			
Model F	1.87			
Model *p*	0.0371			
	**Sig. predictor**	**Estimate**	**SE**	***p***
	Broadband Cz-FR	7.935	3.623	0.0370
	Delta FL-TPR	5.211	2.46	0.0428
	Theta FR-Cz	7.899	3.26	0.0221
	High gamma Oz-FR	8.145	3.61	0.0320

Whole model R^2^ and p-values for the predictive value to FRI for the eyes open resting condition. Parameters for the significant predictive features are also presented.

## Discussion

This study aimed to identify the relationship between resting EEG cortical dynamics and cognitive functioning in school-aged children. Our first main finding showed that regardless of resting condition (eyes open vs. eyes closed), cortical signal complexity as measured by MSE was the same across children with normal to above average cognitive ability. This contradicted our initial hypothesis and contrasts with previous studies, stating that signal complexity increases with the level of ability. Although many studies investigating neuropsychiatric conditions have observed a positive relationship between global intellectual level (FSIQ) and EEG signal complexity^5,19,23^, this did not apply to the participants in this study. A potential explanation lies in the types of participants recruited between studies. Indeed, most previous studies were performed with children that had severe impacts on their levels of functioning (FSIQs below the normal levels), often with genetic etiology. Meanwhile, the participants in the current study were specifically selected to be neurotypical, with no known genetic or neuropsychiatric conditions, and with FSIQs falling within the average to above-average range. Based on the literature and our results, we can hypothesize that brain signal complexity increases with cognitive functioning levels from low to normal levels, reaching a plateau beyond which it may not predict further increases in cognitive functioning. Our effective connectivity analysis results demonstrate that resting cortical dynamics might be more attuned within a healthy population and across this range of cognitive abilities. Indeed, the direction of information flow offers greater insight into the relationship between resting brain dynamics and cognitive functioning than the complexity of cortical area’s signals.

Overall, we observed directed connections between frontal and parietal regions and frontal and occipital regions that were positively related to higher cognitive ability. This agrees with previous findings showing that information flows in a more precise and well-defined direction between fronto-parietal and anterior–posterior brain connections with increasing cognitive ability in children^30^. In accordance with Chen et al.^34^, our results demonstrate that cortical information in high-frequency bands flows more readily from occipital to frontal regions with increasing verbal, visual, processing speed, and general cognitive abilities. Similarly, for these same cognitive functions, low frequencies (delta and theta) information flowed from frontal to parietal regions. These results confirm that fronto-parietal connections are also activated during resting state brain dynamics and corroborate results found in other studies^30,52^. Furthermore, evidence shows that healthy efficient brains show increased locally processed information and decreased distal activity^10,18,21,33^. This pattern of resting connections is described as a small-world network or short-range connections, which provides the optimal balance between dense local clustering of neuronal activity and sparse long-range connections^33,53^. Our results demonstrate an increasing strength of direction for connections between frontal and central regions, frontal regions together, and temporoparietal regions together, which can be related to small-world network connections^54^. Our results convey that even during resting state, neuronal pathways associated with better cognitive performance are actively engaged in a specific direction.

Another main finding of this study is that some differences in dPLI results were observed for specific IQ indexes (i.e. cognitive function). To begin with, directed connectivity information flow in fluid reasoning (FRI) abilities and general intellectual abilities (FSIQ) are very similar. Specifically, no clear directional activity was observed in the eyes closed condition for fluid reasoning (FRI) and general intellectual ability (FSIQ). In the eyes open condition, fronto-central bidirectional activity (theta band) and fronto-parietal (delta band) low-frequency activity were observed. These shared patterns of information flow for fluid reasoning and general intelligence, reflect that these two areas of cognitive functioning are related to the same neuronal pathways during resting state. Of note, research on gifted adults (i.e. adults with above-average performances on psychometric tests of intelligence) has shown that high fluid reasoning skills were predictive of above-average general intelligence scores^55^. Indeed, the authors explained this relationship that fluid reasoning helps build efficient strategies that can increase performance in all other cognitive functions (e.g. verbal, visual, working memory, and processing speed). Given our results, this link potentially holds in children with above-average abilities and explains why similar neural pathways are observed for both these functions.

Out of all the IQ indices, working memory abilities (WMI) was the only one that did not show any relationship with EEG measures (e.g. complexity or connectivity) in both resting conditions (e.g. eyes open or closed). These results show that the direction of the functional connections between the brain regions in resting cortical dynamics does not vary with increasing working memory abilities. This result went against previous findings from our team that found a positive relationship between the response to an overt auditory oddball task and working memory abilities in children^56^. This result reflects that cortical activity is equally bidirectional between brain regions in resting children with normal to above-average working memory abilities. Research has shown that mind-wandering happens during resting conditions, and is related to the amount of cognitive resources (i.e. working memory capacity) an individual has^57,58^. Precisely, under conditions of low cognitive demand (e.g. during resting), a positive relationship exists between increasing working memory abilities and heightened mind-wandering^59^. One can hypothesize that the children in this study with above-average working memory capacity engaged in mind-wandering and had diffused bidirectional patterns of information flow between cortical regions. Notably, we did not probe for mind-wandering thoughts in our experiment, therefore this hypothesis cannot be confirmed. Further studies are needed to fully understand the relationship between working memory abilities and resting brain dynamics.

An additional important observation was that the relationship between cortical dynamics during rest and intellectual potential was more pronounced when children kept their eyes open compared to when they had their eyes closed. More precisely, the results demonstrated that directed connectivity in the eyes open condition correlated more with IQ and its indexes than when the eyes were closed. The eyes-open condition exhibited greater dynamism, reflected by the involvement of a broader range of cortical regions and frequencies, as well as relationships with more IQ indexes when compared to results obtained in the eyes-closed condition. Hence, the eyes-open condition may have been better suited to our EEG metrics. The silent movie used during the eyes-open recordings may have induced some more cortical activation and information flow rather than increasing one rhythm frequency, namely the alpha frequency rhythm unifying the brain dynamics^60^. Eyes closed connectivity results correlated only with one cognitive sub-scale. The verbal ability directed connectivity results were revealed for the alpha frequencies, dominating in frontal to parietal, central to frontal, and bidirectional frontal information flow patterns. The use of a silent movie during the resting condition with eyes open typically enables higher-quality data. While employed to maintain participant engagement and minimize movement, the potential influence of the movie on our results cannot be discounted. Future studies could explore alternative methods, such as employing an engaging fixation cross, to mitigate this effect.

Unrelated to the EEG results, chronological age was positively related to verbal abilities (i.e. VCI). This means that in our cohort, age was a significant predictor of verbal ability. Verbal abilities, as assessed by the Wechsler scales of intelligence, are regarded as part of crystalized skills, which continue to develop with age along with vocabulary and verbal knowledge^61^. Considering that verbal index scores are standardized for age, this result implies that these crystalized abilities develop faster in children with above-average verbal abilities. Further studies are warranted to confirm this finding.

Altogether, these results shed light on the intricate relationship between RS-EEG dynamics and cognitive potential in children, suggesting that specific patterns of cortical information flow may underlie cognitive abilities across various domains. Our study is one of the first to show that the direction of resting brain dynamics is related to the level of cognitive functioning in children with average to above-average intelligence. This research underscores the notion that cognitive abilities are reflected in cortical dynamics even during periods of non-task-related brain activity, offering a potential starting point for further cognitive engagement. Also, directionality analysis of connectivity allowed to add precision to our understanding of the well-known connections related to cognitive ability. The results suggest that theories involving fronto-parietal and anterior–posterior information flow in intelligence may extend to resting recording conditions. Moreover, it is the first to reveal that specific cognitive functions included in general intellectual ability have a distinct relationship with resting brain connectivity. Methodologically, our findings underscore the importance of prioritizing EEG recordings during resting periods with eyes open in studies involving children, given the richness of the data obtained. Future research endeavors should aim to explore whether similar relationships exist with other cognitive functions such as executive functions and attentional processes.

## Data Availability

Datasets generated during and/or analyzed for this study are available through the corresponding author upon reasonable request.
